# Invert emulsions alleviate biotic interactions in bacterial mixed culture

**DOI:** 10.1186/s12934-022-02014-w

**Published:** 2023-01-20

**Authors:** Alexis Dijamentiuk, Cécile Mangavel, Annelore Elfassy, Florentin Michaux, Jennifer Burgain, Emmanuel Rondags, Stéphane Delaunay, Sandie Ferrigno, Anne-Marie Revol-Junelles, Frédéric Borges

**Affiliations:** 1 LIBio, Université de Lorraine, Nancy, France; 2 LRGP, Université de Lorraine, Nancy, France; 3 IECL, Equipe BIGS, INRIA Nancy, Université de Lorraine, Nancy, France

**Keywords:** Invert emulsion, Co-culture system, Competition, Mixed culture, Microbial community, Diversity

## Abstract

The large application potential of microbiomes has led to a great need for mixed culture methods. However, microbial interactions can compromise the maintenance of biodiversity during cultivation in a reactor. In particular, competition among species can lead to a strong disequilibrium in favor of the fittest microorganism. In this study, an invert emulsion system was designed by dispersing culture medium in a mixture of sunflower oil and the surfactant PGPR. Confocal laser scanning microscopy revealed that this system allowed to segregate microorganisms in independent droplets. Granulomorphometric analysis showed that the invert emulsion remains stable during at least 24 h, and that the introduction of bacteria did not have a significant impact on the structure of the invert emulsion. A two-strain antagonistic model demonstrated that this invert emulsion system allows the propagation of two strains without the exclusion of the less-fit bacterium. The monitoring of single-strain cultures of bacteria representative of a cheese microbiota revealed that all but *Brevibacterium linens* were able to grow. A consortium consisting of *Lactococcus lactis* subsp. *lactis* biovar diacetylactis, *Streptococcus thermophilus*, *Leuconostoc mesenteroides*, *Staphylococcus xylosus*, *Lactiplantibacillus plantarum* and *Carnobacterium maltaromaticum* was successfully cultivated without detectable biotic interactions. Metabarcoding analysis revealed that the system allowed a better maintenance of alpha diversity and produced a propagated bacterial consortium characterized by a structure closer to the initial state compared to non-emulsified medium. This culture system could be an important tool in the field of microbial community engineering.

**Supplementary Information:**

The online version contains supplementary material available at 10.1186/s12934-022-02014-w.

## Introduction

Food fermentation processes involve consortia of microorganisms interacting with each other and transforming the matrix into desired final products. Yet, complex starter cultures are composed of a diversity of microorganisms that are mostly cultivated separately before mixing [1]. For obvious economic and logistical reasons, both scientists and industrials are willing to rely on alternatives such as mixed cultures. However, the maintenance of taxonomic or phenotypic diversity in a mixed culture is inherently difficult because of microbial interactions. In particular, competition between microorganisms reduces diversity in the local environment in which it takes place [2]. Several co-culture systems have been designed in order to modulate interactions between microorganisms. Most of these systems rely on the degree of spatial segregation and diffusion of molecules between microorganisms [3]. Sodini et al. designed a method to operate continuous fermentation and inoculation of milk in a bioreactor by entrapping separately four different lactic acid bacteria in carrageenan gel beads [4]. However, this approach leads to the release of bacteria and compounds which ultimately compromise the spatial segregation of microorganisms. Micro-droplets used as bioreactors is now a common approach for the study of bioenergetics, cell-to-cell interactions and co-cultivation of bacteria, and usually relies on the use of microfluidic or millifluidic devices [5]. In principle, such systems reduce the diffusion of inhibitory molecules and disrupt contact-dependent competitive phenomena. Invert emulsions of microbial growth medium prepared with microfluidic devices have also been used to encapsulate micro-organisms in order to enrich cultures with slower-growing strains that would otherwise be excluded by the cultivation process [6, 7]. While this technology enables to precisely control the volume of microreactors and work at high throughput, it is nonetheless inappropriate for large scale amplification of bacteria of interest, notably in the context of agroindustry and complex starter culture production. At these scales, other designs adjusting the degree of interaction between microorganisms have been used. For example, Devanthi et al. have demonstrated the utility of double emulsions (W_1_/O/W_2_) in order to segregate a yeast in the internal W_1_ phase, and a bacterium in the external W_2_ phase. This cultivation system allowed to propagate these micro-organisms while avoiding antagonisms and then to sequentially release them for soy sauce fermentations [8]. Similarly, a double emulsion could also be used to enrich yoghurts with the probiotic strain *Lactobacillus paracasei* without interfering with starter cultures and the fermentation process [9]. However, these studies focused on competition related to exploitation of resources and did not consider antagonisms resulting from interference competition which involves the production of antimicrobial compounds such as bacteriocins [10]. Yet, antimicrobial compounds are widespread in microbial ecosystems [11–14] and their presence might interfere with a wide range of biotechnological applications involving microbiomes [15]. In particular, bacteriocins are ribosomally synthetized peptides with antimicrobial properties allowing their producer to exclude a competitor from a shared ecological niche. These compounds are frequently found in lactic acid bacteria [16, 17] which are of high biotechnological interest for the food and medical industries [18]. It is therefore essential to consider the presence of bacteriocins when designing mixed microbial culture systems.

The aim of the present study was to evaluate the potential of single invert emulsions to alleviate competition during the culture of antagonistic microorganisms and therefore to maintain diversity in a more complex mixed culture. Invert emulsions are typically inter-dispersions of two immiscible liquids wherein droplets of an aqueous phase are dispersed in a continuous oil phase. The investigated invert emulsion system was designed in order to confine microorganisms into droplets of aqueous culture medium; the continuous oil phase would provide a physical barrier to either contact or the transfer of diffusible inhibitors between them.

## Material and methods

### Bacterial cultures

*Carnobacterium maltaromaticum* F2 [19] was isolated by using MCM agar (Trypticase Soy Agar (TSA) (bioMérieux, Marcy-l’Etoile, France) with 6 g L^−1^ of yeast extract (YE), supplemented with 3.5 mg L^−1^ vancomycin, 5 mg L^−1^ gentamicin and 20 mg L^−1^ nalidixic acid) [20] with a sterile loop and incubating the plates at 25 °C for 36 h. Isolates of *Listeria monocytogenes* EGDe were prepared by inoculation of PALCAM agar (Biokar Diagnostics, Paris, France) followed by incubation at 37 °C for 24 h. Colonies were inoculated into fresh Trypticase Soy Broth (TSB) medium (bioMérieux, Marcy-l’Etoile, France) supplemented with 6 g L^−1^ of bacto-yeast extract (YE), bacteria were allowed to grow at 30 °C for 24 h. Aliquots of the resulting culture were supplemented with glycerol to a final concentration of 10% (v/v) and kept at − 80 °C for later use as starter cultures. Other strains used in bacterial consortium cultivation experiments were obtained from various sources, as shown in Table 1. Twenty-four hours before the experiments, separate pre-cultures of each strain were prepared by cultivation in TSBYE for 24 h at their optimal growth temperature (Table 1).

**Table 1:** Bacterial strains used in this study

Strain	Growth temperature (°C)
*Lactococcus lactis* subsp. *lactis* biovar diacetylactis	25
*Streptococcus thermophilus*	42
*Leuconostoc mesenteroides*	25
*Staphylococcus xylosus*	30
*Carnobacterium maltaromaticum* F2	30
*Lactiplantibacillus plantarum* WHE92	30
*Brevibacterium linens*	25
*Listeria monocytogenes* EGDe	37
*Escherichia coli* DH5-α pGLO	37

Just prior to the experiments, cultures were prepared for each strain by diluting the pre-cultures into fresh medium until a final bacterial density of 10^6^ CFU mL^−1^, unless otherwise specified. Mixed cultures of *C. maltaromaticum* F2 and *L. monocytogenes* EGDe were prepared from individual pre-cultures to obtain a cell-to-cell ratio of 1:1. Non-emulsified pure and mixed cultures were incubated 24 h at 30 °C to serve as control in emulsified cultivation experiments. For bacterial consortium cultivation experiments, mixed cultures of the aforementioned bacteria were prepared by mixing equal volumes of pre-cultures in fresh TSBYE medium, final volume 20 mL, so as to obtain a total density of 10^6^ CFU mL^−1^. Classical cultures were produced by using 15 mL from the new mixture while 3 mL were used for producing invert emulsion cultures. Mixed cultures were then incubated at 30 °C for 72 h. In parallel, each strain was cultivated separately in both systems. After 72 h incubation, equal volumes of cultures in classical setup and invert emulsion were pooled and used as controls simulating the absence of interaction in classical setup or invert emulsion, respectively. Propagation of a bacterial consortium was performed in quadruplicate; pure culture experiments were performed in triplicate. *Escherichia coli* DH5-α was transformed with the pGLO plasmid according to Sambrook and Russell [21], allowing the bacterium to express Green Fluorescent Protein (GFP) when cultivated in the presence of arabinose. This strain was maintained on TSAYE containing 100 µg mL^−1^ ampicillin and 33 µg mL^−1^ d,l-arabinose.

### Bacterial enumeration

Cultures were ten-fold serially diluted in tryptone-salt solution (Biokar Diagnostics, Paris, France) with 2% Tween^®^ 80 (Merck KGaA, Darmstadt, Germany) with thorough vortexing. Serial dilutions were used to inoculate agar medium Petri dishes in order to count colonies for enumeration after cultivation. Non-specific enumeration was performed on TSAYE, while specific enumerations in competition experiments were performed on the selective agar media MCM and PALCAM for *C. maltaromaticum* F2 and *L. monocytogenes* EGDe, respectively. TSAYE and MCM plates were incubated at 25 °C for 36–48 h for *C. maltaromaticum* F2, while TSAYE and PALCAM plates were incubated at 37 °C for *L. monocytogenes* EGDe. Given the proportion of aqueous medium in the invert emulsion, raw cell numbers in emulsified cultures were corrected by a factor of 5. Experiments were performed in triplicate.

### Growth kinetics upon 24 h

Growth parameters were obtained by using R [22] and the R package *growthrates* [23]. The Baranyi growth model was fitted to enumeration data [24, 25]. Box constraints in the Baranyi model, namely initial N value (y0), maximum growth rate (µ_max_), carrying capacity (K) i.e., maximal population density and initial physiological state $h0=\mu {\text{max}} \times lag$ were extracted from prior observation of the dataset and applied in order to optimize the fitting before growth parameters were estimated. Generation time was obtained by the formula $\ln \left( 2 \right) \div \mu_{\max }$. Lag time values were obtained by dividing the h0 parameter of the model by µ_max_. Experiments were done in triplicate.

### Invert emulsions

Small-scale invert emulsions were prepared by dropwise addition of 3 mL sterile or inoculated TSBYE into a mix of 11.625 g refined sunflower oil (Système U, Rungis, France) and 375 mg polyglycerol polyricinoleate (PGPR) (Palsgaard A/S, Juelsminde, Denmark), i.e., PGPR 2.5% of the total weight, in a 40 mL, 30 mm diameter vial (CEB, Angers, France). Emulsification was ensured by a magnetic stirrer (IKA-Werke GmbH & Co. KG, Staufen, Germany) using a polygonal stir bar with pivot ring (L: 20 mm; Ø 6 mm) at 400 rpm and 20 °C, during 5 min counting from the moment the last drop of aqueous phase was added. After emulsification, the invert emulsions were transferred into a 15 mL CELLSTAR^®^ centrifugation tube (Greiner Bio-One International GmbH, Kremsmünster, Austria) and placed on a Stuart SB-2 tube rotator (Cole-Parmer LLC, Vernon Hills, USA) at 20 rpm, the tube holder having a 45° tilt in order to avoid both sedimentation and excessive shear of the samples. The samples were incubated at 30 °C under continuous rotation to allow bacterial propagation.

Bioreactor-scale emulsification took place in aseptic conditions in a 1 L bioreactor (GPC, Périgny, France). It was carried out at 30 °C by addition under stirring at 250 rpm with a 6-bladed Rushton propeller of 120 mL inoculated TSBYE in a mix of 465 mL sunflower oil and 15 g PGPR. The stirring speed was reduced to 175 rpm after 20 min in order to maintain droplets in suspension for the rest of the experiment.

The breaking of 1.5 mL of invert emulsion was carried in several steps according to a protocol adapted from Bachmann et al*.* [6]. First, the oil phase was removed after centrifugation of the sample at 5000 rcf for 10 min. Then, 300 µL 1*H*,1*H*,2*H*,2*H*-perfluoro-1-octanol (Sigma-Aldrich Corporation, Saint Louis, MO, USA) were added and the sample was shaken gently over 15 min. Finally, the mix was let still until phase separation.

### Particle size distribution

Particle size distribution was determined by dynamic image analysis using the QICPIC granulomorphometer with LIXELL dispersion system (Sympatec GmbH, Clausthal-Zellerfeld, Germany) coupled with the QICPIC image analysis software. This technique is based on the acquisition of high-resolution pictures of particles (herein droplets) within a liquid solution [26]. Just before analysis, 0.5 mL of emulsion was gently diluted into 50 mL sunflower oil. The dilution was then injected into the 0.2 mm cuvette using a syringe. Measurement time was 30 s per run and each measurement was done in triplicate. Diameters were automatically sorted into 30 classes ranging from 3 to 750 µm. Particle size distributions were expressed as the volumetric density distribution (q3lg) for each class of diameter. The span values, normalizing the width of the distribution relatively to the median value *d*_50_, were calculated as:

$$\mathrm{span}= \frac{{d}_{90}- {d}_{10}}{{d}_{50}}$$

where *d*_10_ and *d*_90_ are the first and ninth deciles and d_50_ is the median of the particle size distribution.

### Confocal laser scanning microscopy (CLSM)

The day before CLSM observations, fluorescent isolates of *Escherichia coli* DH5-α pGLO were transferred into 10 mL TSBYE supplemented with ampicillin 100 µg mL^−1^ and D,L-arabinose 33 µg mL^−1^ for overnight preculture at 37 °C under vigorous agitation. On the day of the experiment, this culture was emulsified. Fifty microliters of the freshly prepared emulsified culture were collected and added to 950 µL sunflower oil. Ten microliters of the lipophilic stain Nile Red (Sigma-Aldrich Corporation, Saint-Louis, MO, USA) were added to the mix thereafter homogenized by mild inversions. Twenty-five microliters of the mix were deposited on a microscope slide to which the coverslip was then sealed with lacquer. Imaging was performed on a TCS SP5-X-AOBS confocal microscope with white light laser (Leica Microsystems GmbH, Wetzlar, Germany). Magnification was 100× with a numeric zoom factor of 5. Excitation and emission wavelengths λ_ex_ and λ_em_ for the observation of the bacteria and continuous phase were determined by prior spectral scanning (Additional file 1: Fig. S1). Retained values were λ_ex_(GFP) = 481 nm, λ_em_(GFP) = 499–517 nm and λ_ex_(Nile Red) = 539 nm, λ_em_(Nile Red) = 559–613 nm. Images were acquired in sequential mode to avoid fluorescence crosstalk, then enhanced and exported using LAS X software (Leica).

### Inhibition assays on agar

Twenty-five microliters of supernatant obtained from non-emulsified cultures or broken emulsified cultures of *Carnobacterium maltaromaticum* F2 were heated to 80 °C for 30 min in order to kill remaining bacteria. The treated supernatants were then added into wells dug in soft TSAYE plates pre-inoculated with a pre-culture of *L. monocytogenes* EGDe so as to obtain a final OD_590_ of 0.01. Plates were stored at 4 °C during 24 h to allow diffusion of the deposits, then incubated 24 h at 30 °C to allow growth of L. monocytogenes EGDe. This method was adapted from the agar well diffusion assay method described by Holder and Boyce [27].

### DNA extraction and amplification of the 16S rRNA V4 region and sequencing

The DNA extraction was performed in two steps using the NucleoSpin Food^®^ kit (Macherey–Nagel, Düren, Germany). Briefly, 1.5 mL of bacterial suspension were centrifugated at 10,000 rcf during 10 min and the supernatant was discarded. The pellet was then suspended in 550 µL 65 °C preheated lysis buffer, to which 10 µL of 10 mg mL^−1^ proteinase K were added. The homogenized mixture was then incubated at 65 °C for 3 h, before 10 µL of 20 mg mL^−1^ RNase and between 200 and 300 mg of sterile, UV-treated glass beads (150–212 µm diameter) were added. The new mixture was then shaken at 3200 rpm on a Vortex Génie 2 horizontal agitator (Scientific Industries, New York, USA) for 1 h at room temperature before centrifugation at 10,000 rcf for 10 min. The supernatant was then used for DNA purification according to the manufacturer’s instructions.

Polymerase chain reaction (PCR) targeted the v4 region of the 16S rRNA gene, using three variants of the forward 515F primer (10 µM) at equal volumes, and the reverse 806R primer (10 µM) (Table 2) [28].

**Table 2:** Primers with adapters used for amplification of bacterial 16S rRNA (V4) gene and sequencing

Forward	515F-1	TCGTCGGCAGCGTCAGATGTGTATAAGAGACAGNNNNNN**GTGCCAGCMGCCGCGGTAA**
	515F-2	TCGTCGGCAGCGTCAGATGTGTATAAGAGACAGNNNNN**GTGCCAGCMGCCGCGGTAA**
	515F-3	TCGTCGGCAGCGTCAGATGTGTATAAGAGACAGNNNN**GTGCCAGCMGCCGCGGTAA**
Reverse	806R	GTCTCGTGGGCTCGGAGATGTGTATAAGAGACAGNNNNNN**GGACTACHVGGGTWTCTAAT**

The primer sequence hybridizing to DNA appears in bold characters

Five microliters of 20 ng µL^−1^ DNA extract were transferred into 45 µL PCR mix containing 37.8 µL DNA-grade water, 5 µL AccuPrime™ Buffer II (Invitrogen, Carlsbad, USA), 1 µL of each primer at 10 µM, 0.2 µL AccuPrime™ *Taq* DNA polymerase High Fidelity. After an initial denaturation step at 94 °C for 3 min, 35 PCR cycles were performed as follows: 10 s denaturation at 94 °C, 45 s hybridization at 50 °C, 1 min 30 s elongation at 68 °C. The duration of the final elongation step was 10 min. Miseq V2 Illumina sequencing was conducted by ADNid (Monferriez-sur- Lez, France), providing 2 × 250 bp reads.

### Sequence analysis

The processing of the sequencing reads was conducted using FROGS tools [29] on the Galaxy Migale platform. Bacterial 16S rRNA (V4 region) paired-end reads were merged with a maximum mismatch rate of 10% in the overlap region. Denoising consisted of removing reads that did not match the expected length (i.e., between 240 and 300 bp) or containing ambiguous bases (N). After dereplication, clustering of sequences was conducted with the fastidious option in SWARM, a single-linkage clustering algorithm using a local clustering threshold (aggregation distance of 1 in the present work) [30]. Chimeras were removed and only the 6 most abundant OTUs were kept. Taxonomic affiliation was performed by Blast against the EZBioCloud 16S database [31]. The abundance table was then imported into R [22] for further analysis. Alpha and beta diversity analyses were conducted using tools from the ‘vegan’ R package [32]. For beta diversity, Bray–Curtis distances were calculated with the ‘avgdist’ function, using rarefaction at the minimal sequencing depth (31,444 sequences) upon 100 iterations. Hierarchical clustering of samples was conducted by processing the rarefied distance matrix with the built-in ‘hclust’ function using Ward’s method for agglomeration [33].

### Statistical analysis

Tests for statistical significance were performed on at least three repetitions using R [22]. Experimental data obtained in this study were analyzed using a two-way analysis of variance (ANOVA) using a fixed effects model, followed by Tukey’s HSD test. In the droplet size distributions of the invert emulsions, factors involved were the presence or absence of bacteria, and the incubation of invert emulsions (0 h or 24 h incubation). In bacterial enumerations, factors were the cultivation system used (classical system or invert emulsion) and the incubation (0 h or 24 h incubation). In community cultivation experiments, differences in Shannon diversity index between groups of samples were tested using one-way ANOVA, followed by a Tukey’s HSD test. Results were expressed as means ± SEM (Standard Error of the Mean). Differences were considered to be statistically significant when *P* value was inferior to 0.05. In the figures, means ± SEM followed by a different superscript letter indicate a significant difference (*P* < 0.05).

## Results

### Structure of the invert emulsion

**Fig. 1:**
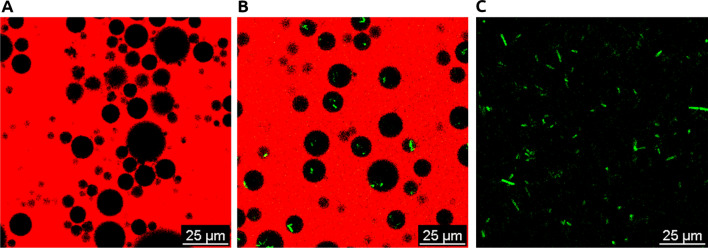
Confocal laser scanning microscopy image of emulsified and non-emulsified cultivation systems. **A** Invert emulsion devoid of bacteria; **B** emulsified culture of GFP producing bacterium *E. coli* DH5-α pGLO; **C** non-emulsified culture of *E. coli* DH5-α pGLO. Black: culture broth; red: Nile Red staining the oil-continuous phase; green: GFP

An invert emulsion free of bacteria was produced by dispersing TSBYE culture medium in a mixture of sunflower oil and the surfactant PGPR. The observation of the emulsion by CLSM confirmed that the system was an invert emulsion as the continuous phase was labelled by the Nile red dye (Fig. 1A). The black circular droplets represented the emulsified phase containing the culture medium.

**Fig. 2:**
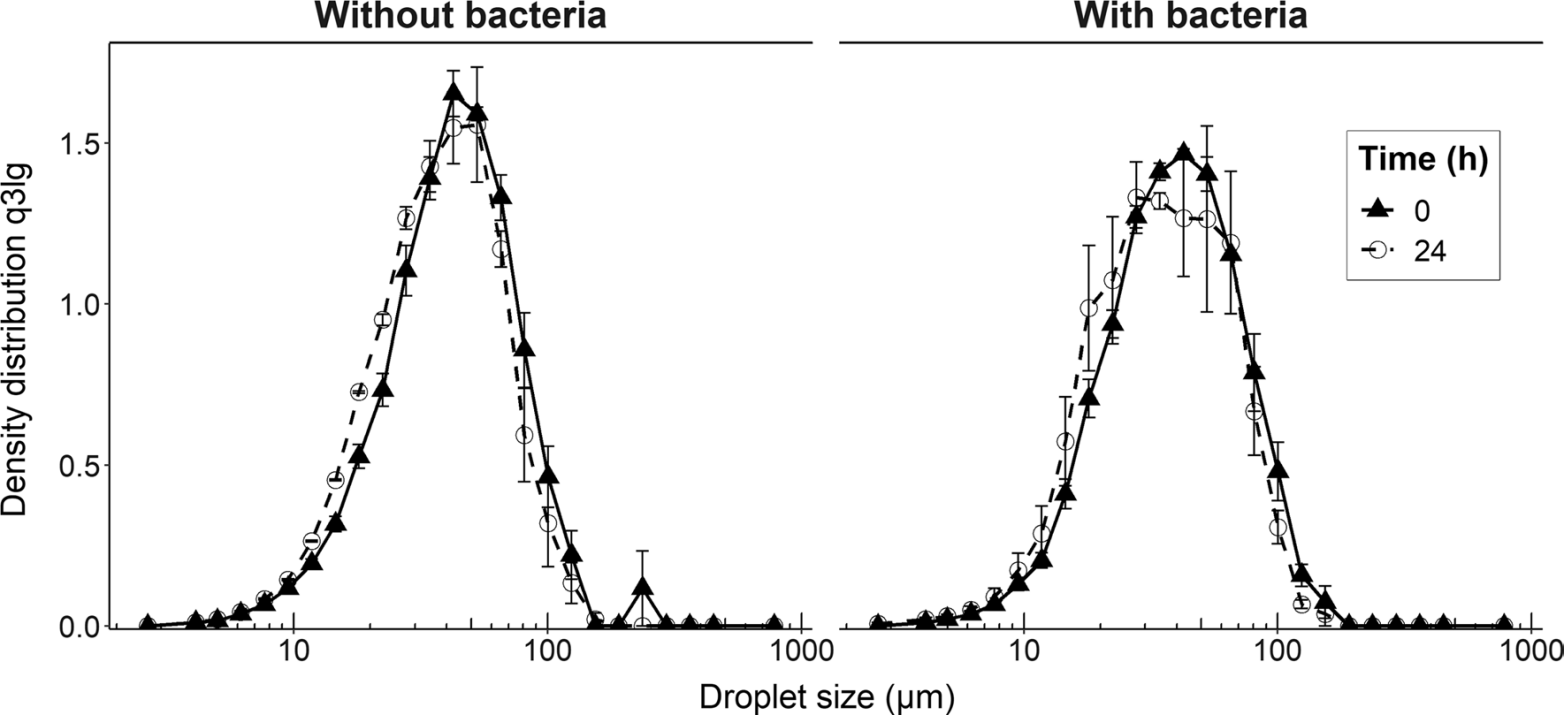
Particle size distribution obtained by granulomorphometric analysis. Logarithmic density volumetric distribution q3lg without bacteria (left) and with bacteria (right), respectively. Solid line, filled triangle: after emulsification; dashed line, empty circle: after 24 h incubation. Error bars represent the SEM

Granulomorphometry allowed to evaluate droplet size distribution in the sample before and after 24 h incubation at 30 °C. Granulomorphometric analysis of freshly prepared emulsions revealed that 80% of the dispersed phase was contained in droplets of d_10_ = 18.5 ± 0.5 µm to d_90_ = 84.1 ± 6.6 µm diameter, the median diameter in the volumetric distribution was d_50_ = 43.1 ± 1.7 µm (Fig. 2, left).

After 24 h incubation, 80% of the volume was contained in droplets whose diameters were comprised between d_10_ = 16.5 ± 0.1 µm and d_90_ = 74.7 ± 5.6 µm (d_50_ = 38.2 ± 0.8 µm) (Fig. 2, left). The width of the distribution, described by span, was 1.52 ± 0.08 after emulsification and did not change after 24 h incubation (1.52 ± 0.12). Droplet size changes in the invert emulsion which could have been occurred by Ostwald ripening or coalescence would have led to a displacement of the distribution towards greater diameters, yet such displacement was not observed. These results show that the obtained invert emulsion was sufficiently stable during 24 h at 30 °C. Considering that most of the volume of the dispersed phase was comprised in droplets whose sizes were deemed compatible with the hosting of bacteria, the bacterium *Carnobacterium maltaromaticum* F2 was then introduced in the culture broth before emulsification. After emulsification, most of the volume was contained in droplets whose diameter was comprised between d_10_ = 17.3 ± 0.7 µm and d_90_ = 81.0 ± 4.2 µm (d_50_ = 39.3 ± 1.5 µm) (Fig. 2, right). Similarly, the bulk of the volume was contained in droplets from d_10_ = 15.9 ± 1.8 µm to d_90_ = 73.1 ± 2.2 µm in diameter (d_50_ = 35.1 ± 4.5 µm) after 24 h incubation (Fig. 2, right). Span values for invert emulsions containing bacteria were approximately 1.62 ± 0.04 after emulsification and 1.68 ± 0.18 after 24 h incubation. Thus, neither d_50_ nor span were significantly affected by introduction of bacteria or incubation (*P* value ≥ 0.05). In a similar manner, an invert emulsion was prepared with a culture of the aero-anaerobic bacterium *Escherichia coli* DH5-α expressing a green fluorescent protein (GFP) with the aim of localizing bacteria in the system using CLSM. It showed that the fluorescent bacteria were confined in these droplets, therefore spatially segregated by the continuous phase (Fig. 1B) as opposed to a non-emulsified culture where all bacteria shared the same space (Fig. 1C). As the introduction of bacteria had a negligible effect on both structure and stability of the system, the invert emulsion system was considered suitable for bacterial cultivation assays.

### Bacterial culture in invert emulsion

**Fig. 3:**
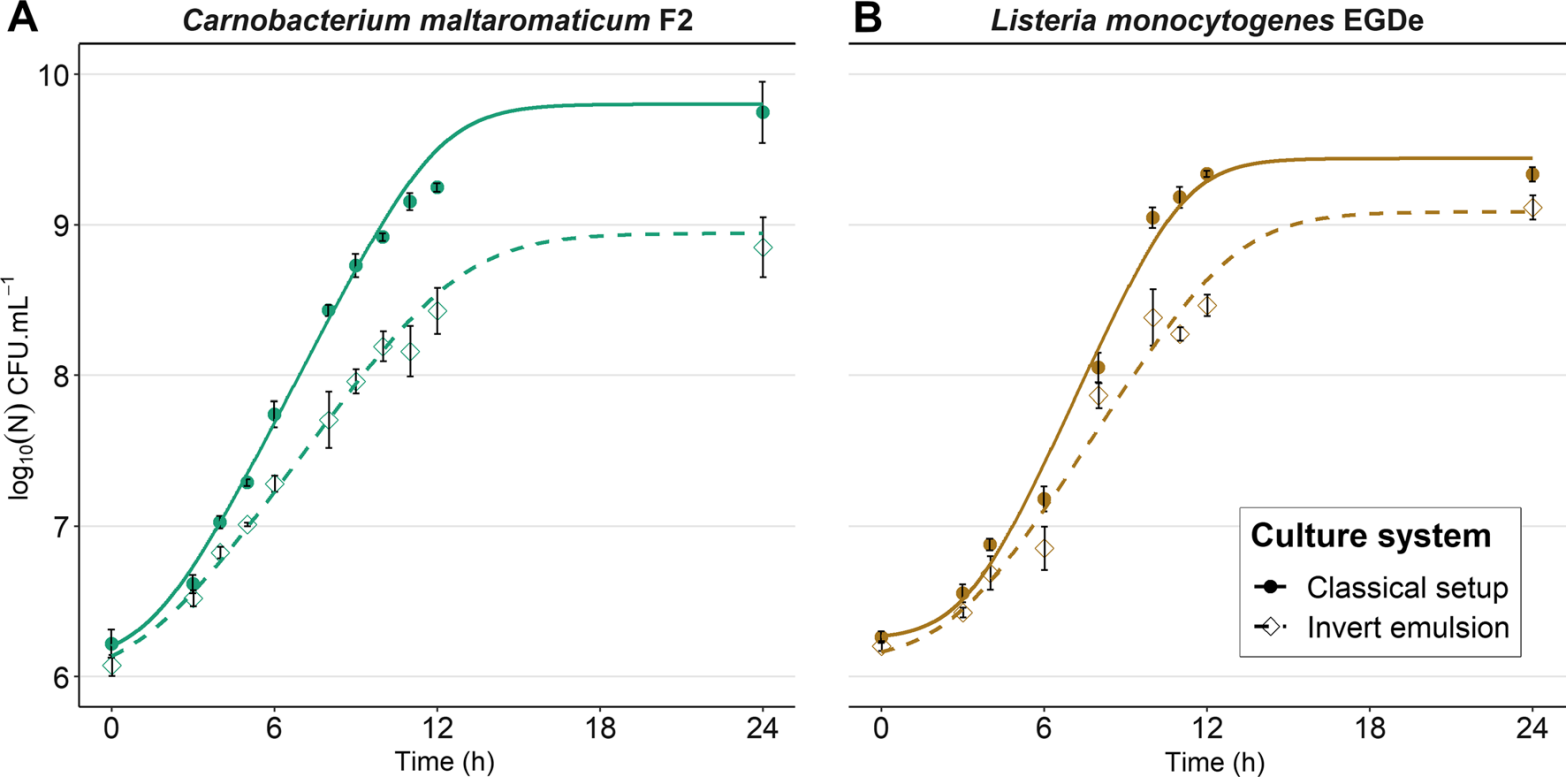
Effect of the culture system on the growth kinetics of **A** *C. maltaromaticum* F2, and **B** *L. monocytogenes* EGDe. Solid and dashed lines represent Baranyi growth models fitted on the data. Filled circles, solid lines: classical setup; empty diamonds, dashed lines: invert emulsion. Error bars represent the SEM

In order to investigate the impact of invert emulsion on competition, a model couple of antagonistic bacteria was constituted. The model consists of *Carnobacterium maltaromaticum* F2 which produces a Bacteriocin-LIke Substance (BLIS) able to inhibit the growth of *Listeria monocytogenes* EGDe, as illustrated by the presence of an inhibition halo on a double-layer agar assay (Additional file 1: Fig. S2). Each bacterium was first cultivated separately in invert emulsion and in non-emulsified medium (i.e., classical setup). The mean generation time of *C. maltaromaticum* F2 reached approximately 52 ± 4 min in non-emulsified culture while it reached approximately 70 ± 3 min in invert emulsion (*P* value < 0.05) (Fig. 3A).

The lag time was similar in both culture systems: 108 ± 8 min in classical culture and 109 ± 3 min in invert emulsion. The mean carrying capacity in invert emulsion was 8.86 ± 0.19 log_10_ CFU mL^−1^, significantly lower than in a classical system where it reached 9.69 ± 0.21 log_10_ CFU mL^−1^ (*P* value < 0.05). Similar differences were observed between culture systems with *L. monocytogenes* EGDe (generation time of approximately 45 ± 1 min in non-emulsified culture and 63 ± 5 min in invert emulsion) but were not statistically significant (*P* value ≥ 0.05) (Fig. 3B), as well as the differences in lag time (193 ± 2 min in classical culture and 169 ± 31 min; *P* value ≥ 0.05). The carrying capacity was significantly lower in invert emulsion (9.07 ± 0.09 log_10_ CFU mL^−1^) than in a classical system (9.44 ± 0.03 log_10_ CFU mL^−1^) (*P* value < 0.05). These results show that bacteria can grow in an invert emulsion. In addition, invert emulsion led to a lower carrying capacity of the medium and a lower growth rate for *C. maltaromaticum* F2.

**Fig. 4:**
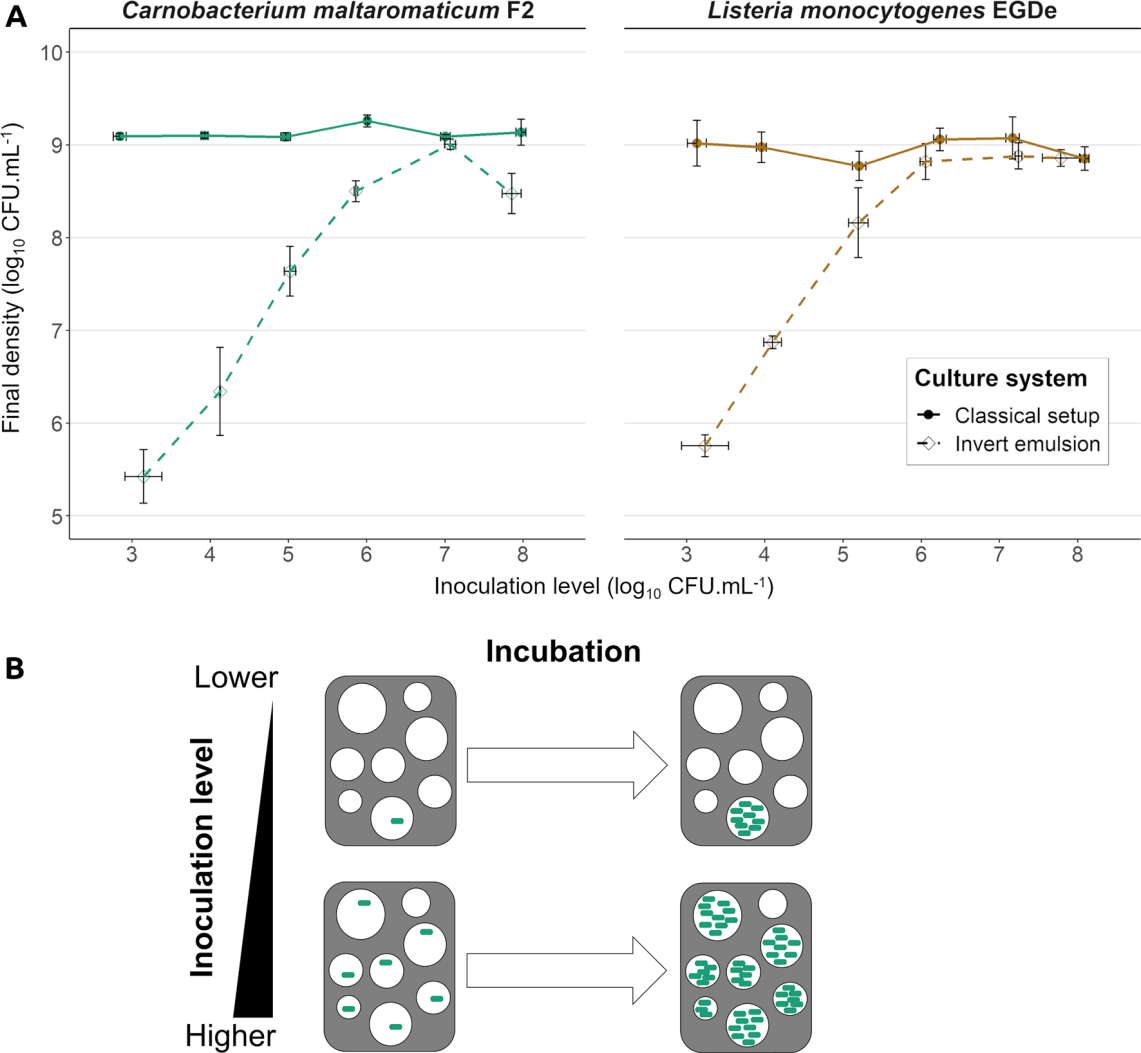
**A** Effect of varying initial densities on the growth dynamics of separately cultivated bacteria. Green: *C. maltaromaticum* F2; brown: *L. monocytogenes* EGDe; filled circles, solid lines: classical setup; empty diamonds, dashed lines: invert emulsion. Error bars represent the SEM. **B** Suggested mechanism explaining the sensitivity of growth dynamics to the inoculation levels in an invert emulsion culture

The impact of inoculation levels on growth capacity was subsequently investigated by cultivating *C. maltaromaticum* F2 and *L. monocytogenes* EGDe separately with different initial densities. In non-emulsified culture, the final level of bacterial densities was approximately the same and reached 10^9^ CFU mL^−1^ for both bacteria (Fig. 4A, left and right, Additional file 1: Table S1).

Consequently, and as expected, the inoculation level had a low impact on the final densities in a non-emulsified medium. By contrast, in invert emulsified medium the final density of *C. maltaromaticum* F2 increased when the inoculum increased from 10^3^ to 10^6^ CFU mL^−1^ and reached the same amount as in a classical culture at the 10^7^ CFU mL^−1^ inoculation level (Fig. 4A, left, Additional file 1: Table S1). At the highest inoculation level (10^8^ CFU mL^−1^), the bacterial density slightly decreased (Fig. 4A, left, Additional file 1: Table S1). In terms of amount of growth, given by the final density divided by the initial density, the increase of *C. maltaromaticum* F2 populations in invert emulsion averaged 2.4 log_10_ regardless of the inoculation level between 10^3^ and 10^6^ CFU mL^−1^. The amount of growth then decreased to 1.9 log_10_ when the invert emulsion was inoculated with 10^7^ CFU mL^−1^, and to 0.6 log_10_ when the inoculum was of 10^8^ CFU mL^−1^. Similarly, the final density of *L. monocytogenes* EGDe increased with the initial density when comprised between 10^3^ and 10^6^ CFU mL^−1^, then reached its maximum approximating 10^9^ CFU mL^−1^ from 10^6^ CFU mL^−1^ inoculation level (Fig. 4A, right, Additional file 1: Table S1). More precisely, the amount of growth of *L. monocytogenes* EGDe in invert emulsion averaged 2.8 log_10_ from the 10^3^ to 10^6^ CFU mL^−1^ inoculation levels. It then decreased to 1.6 log_10_ at the 10^7^ CFU mL^−1^ inoculation level and finally to approximately 1.1 log_10_ at the highest inoculation level. This experiment demonstrates that the growth dynamics of bacteria cultivated in invert emulsion remains constant as long as the initial density remains inferior to 10^6^ to 10^7^ CFU mL^−1^. When this threshold is reached, growth dynamics diminish and the final densities do not exceed those retrieved in a classical system. Therefore, bacteria should be inoculated under this threshold in order to preserve maximal growth dynamics in invert emulsion. Overall, these results suggest that the invert emulsion culture system is sensitive to the inoculation level. Especially, higher inoculation levels lead to higher final densities as the occupancy rate of droplets increases until it reaches its limits. The suggested behavior is illustrated in Fig. 4B.

### Impact of invert-emulsion culture on interference competition

**Fig. 5:**
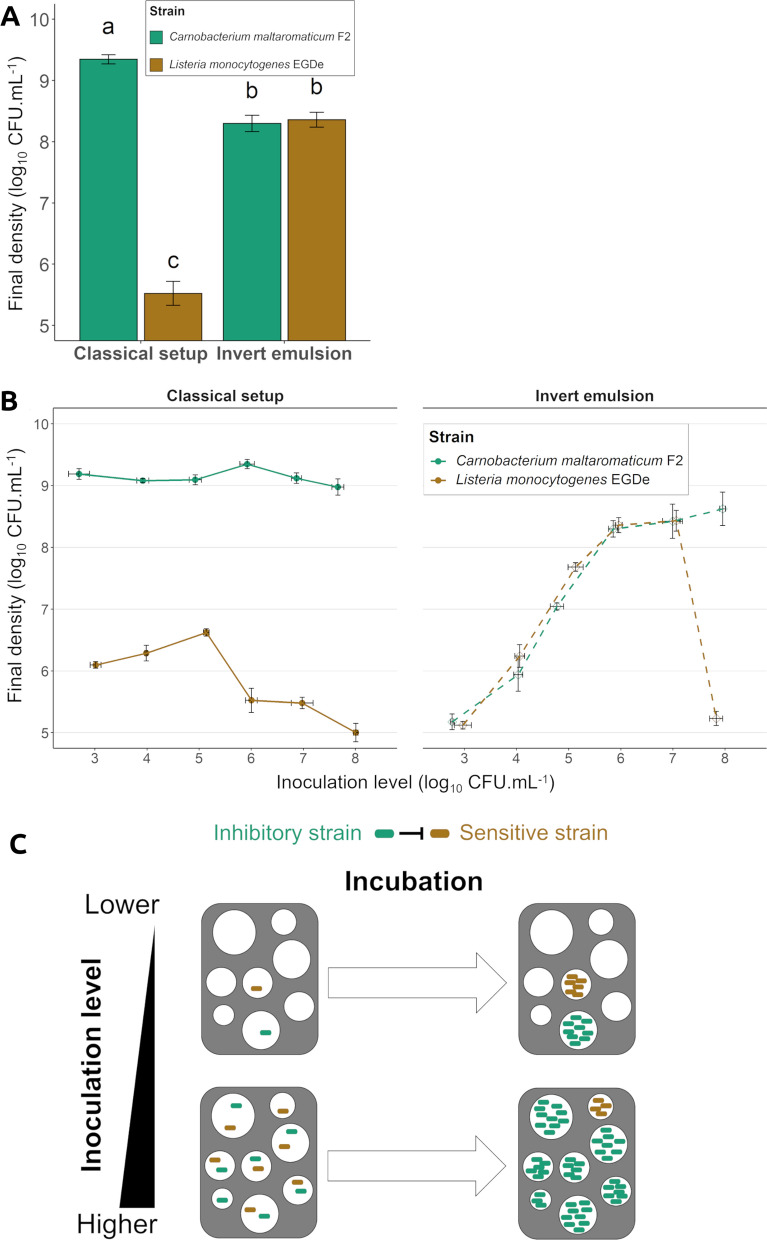
**A** Effect of the culture system on competition between antagonistic strains in a mixed culture, bacteria were co-inoculated with an initial density of 10^6^ CFU mL^−1^. **B** Effect of varying initial densities on competition between antagonistic strains in a mixed culture. **C** Suggested bacterial segregation mechanism leading to the alleviation of competition and sensitivity of this property to inoculation levels. Green: *C. maltaromaticum* F2; brown: *L. monocytogenes* EGDe; filled circles, solid lines: classical setup; empty diamonds, dashed lines: invert emulsion. Error bars represent the SEM; a different superscript letter indicate a statistically significant difference (*P* value < 0.05)

In order to study the effect of invert emulsions on competition between *C. maltaromaticum* F2 and *L. monocytogenes* EGDe, both strains were co-cultivated with an initial cell-to-cell ratio of 1:1. Bacterial densities were separately enumerated by using selective media. When bacteria were co-inoculated in a classical system at 10^6^ CFU mL^−1^, *C. maltaromaticum* F2 reached approximately 10^9^ CFU mL^−1^ (Fig. 5A, left, Additional file 1: Table S2).

This is similar to the growth of the bacterium observed in a pure classical culture at this inoculation level (Fig. 4A, left, Additional file 1: Table S1). As expected, *L. monocytogenes* EGDe densities were markedly low (5.5 ± 0.2 log_10_ CFU mL^−1^) at the end of a classical mixed culture (Fig. 5A, left, Additional file 1: Table S2). However, when the antagonistic strains were co-cultivated in invert emulsion, both displayed growths up to approximate final densities of 10^8^ CFU mL^−1^ and growth dynamics approximating 2.3 log_10_ (Fig. 4A, right, Additional file 1: Table S2). These outcomes are consistent with the final densities and growth dynamics observed in pure cultures of each strain in invert emulsion at this inoculation level (Fig. 4A, left and right, Additional file 1: Table S1). These results therefore depict the absence of competition in invert emulsion, invert emulsions thus alleviate the effects of competition between antagonistic strains. As the inoculation level impacted the growth dynamics in pure cultures, we further investigated the effect of initial bacterial densities on competition. In classical mixed culture and regardless of the inoculation levels, final densities of *C. maltaromaticum* F2 reached 9 log_10_ CFU mL^−1^ while those of *L. monocytogenes* EGDe never exceeded approximately 6.6 log_10_ CFU mL^−1^ (Fig. 5B, left, Additional file 1: Table S2). As expected, the inhibitor strain therefore dominated the sensitive strain in classical mixed culture. In invert emulsion, the final density of the inhibitor strain *C. maltaromaticum* increased with the inoculation level and growth dynamics were relatively constant (approximately 2.3 log_10_ on average), until a threshold was reached at 10^6^ CFU mL^−1^ inoculation level, above which the density only slightly increased (Fig. 5B, right, Additional file 1: Table S2). This shows that *C. maltaromaticum* F2 was not affected by the introduction of *L. monocytogenes* EGDe in invert emulsion. In invert emulsion, population densities of the sensitive strain *L. monocytogenes* EGDe presented an analogous trend of increasing densities and almost steady growth dynamics (approximately 2.3 log_10_ on average) for inoculation levels from 10^3^ to 10^6^ CFU mL^−1^. Final densities reached a maximum (8.4 log_10_ ± CFU mL^−1^) at 10^6^–10^7^ CFU mL^−1^ inoculation levels (Fig. 5B, right, Additional file 1: Table S2). This shows that when bacteria were co-inoculated between 3 and 7 log_10_ CFU mL^−1^, the invert emulsion prevented *L. monocytogenes* EGDe from inhibition by the competitor. However, at the highest inoculation level (10^8^ CFU mL^−1^), the density of the sensitive strain decreased to approximately 10^5^ CFU mL^−1^ which corresponded to a 2.6 log_10_ fold decrease (Fig. 5B, right, Additional file 1: Table S2). This result contrasted with that of a pure culture of *L. monocytogenes* EGDe in invert emulsion, where the final density was approximately 10^9^ CFU mL^−1^ (Fig. 4A, right, Additional file 1: Table S1) and was similar to that of a mixed culture in a classical system (Fig. 5B, left, Additional file 1: Table S2). This result shows that competition occurred in the invert emulsion system as it occurred in a classical cultivation system when bacteria were co-inoculated at 10^8^ CFU mL^−1^. The observed competitive phenomenon was likely due to the co-localization of competitors within single droplets in invert emulsion that resulted from the high inoculation level involved, as represented in Fig. 5C. These results demonstrate that the inoculation level for a binary co-culture should not exceed 10^7^ CFU mL^−1^ in order to benefit from the competition-alleviating properties of invert emulsions.

A control experiment was performed in order to check that the model strain *C. maltaromaticum* F2 was able to produce diffusible inhibitory compound under cultivation in emulsified medium. For this purpose, *L. monocytogenes* EGDe was exposed to the aqueous phase from an emulsified culture of *C. maltaromaticum* F2 in an agar assay (Additional file 1: Fig. S4A). Antimicrobial activity was observed around wells containing aqueous phases of emulsified cultures of *C. maltaromaticum* F2 showing that cultivation in invert emulsion did not prevent the bacterium from producing diffusible antimicrobial substances against *L. monocytogenes* EGDe (Additional file 1: Fig. S4B) as it did in classical culture (Additional file 1: Fig. S4A). In addition, a third well contained an emulsion wherein *C. maltaromaticum* F2 had grown, without further treatment. The absence of inhibition zone around this deposit showed that the invert emulsion prevented the diffusion of inhibitory substances into the agar containing *L. monocytogenes* EGDe (Additional file 1: Fig. S4C).

Altogether, these results demonstrated that the invert emulsion culture system limited the effects of interference competition in a mixed culture.

### Scaling-up the invert emulsion cultivation system

A scale-up (40-fold) the invert emulsion culture system was performed. To do so, we used a bioreactor of 1 L capacity to produce 600 mL of emulsified culture medium. The particle size distribution was similar to small-scale emulsions with an initial d_50_ = 39.2 ± 0.3 µm and a span of 1.27 ± 0.01 in non-inoculated invert emulsions. After 24 h incubation, the distribution was significantly displaced towards smaller droplets (d_50_ = 24.2 ± 0.1 µm) and was less polydisperse (span = 1.12 ± 0.01) (*P* value < 0.001) (Additional file 1: Fig. S3, left). The introduction of bacteria significantly displaced the distribution towards smaller droplets (d_50_ = 32.4 ± 0.2 µm) and monodispersity (span = 1.22 ± 0.01) (*P* value < 0.001). Incubation significantly decreased the median diameter of droplets (d_50_ = 22.9 µm, *P* value < 0.001) and the polydispersity of the invert emulsion (span = 1.08 ± 0.01, *P* value < 0.001) (Additional file 1: Fig. S3, right). After checking that *C. maltaromaticum* F2 and *L. monocytogenes* EGDe grew in bioreactor, these strains were then co-inoculated at 10^6^ CFU mL^−1^. As expected, the classical culture was highly favorable for *C. maltaromaticum* F2 whose densities reached 8.5 ± 0.1 log_10_ CFU mL^−1^ after 24 h incubation. In contrast, *L. monocytogenes* EGDe reached 6.1 ± 0.1 log_10_ CFU mL^−1^. In invert emulsion, *C. maltaromaticum* F2 and *L. monocytogenes* EGDe reached 8.2 ± 0.1 and 8.1 ± 0.3 log_10_ CFU mL^−1^ respectively. The antagonistic model thus displayed competition in non-emulsified culture but not in invert emulsion, in a similar way to the small-scale condition. This demonstrates that the invert emulsion system can be used at larger scales to reduce competition between a couple of antagonistic bacteria.

### Effect of the invert emulsion system on the growth of different strains

**Fig. 6:**
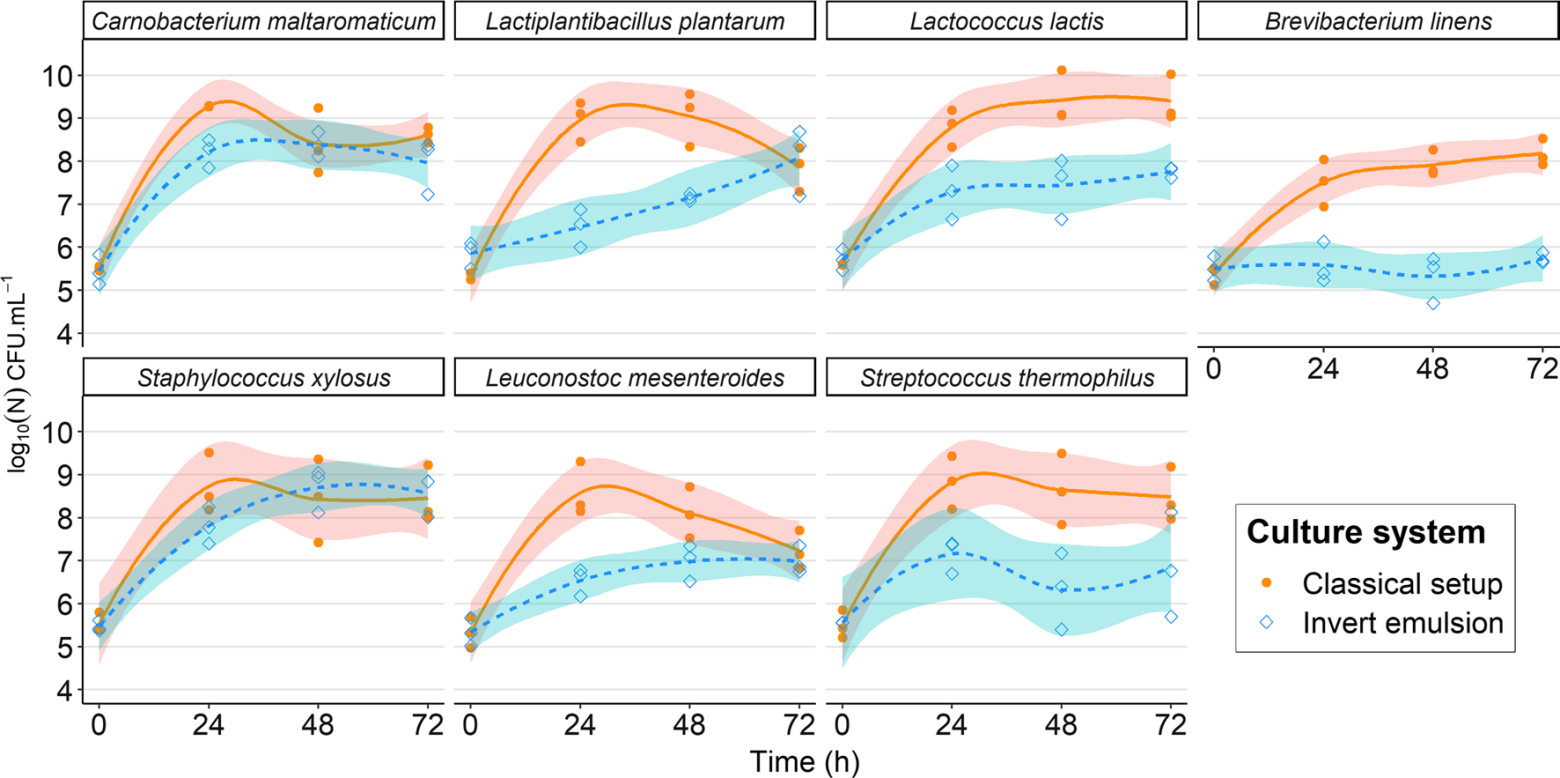
Growth kinetics of selected bacterial strains in classical setup or invert emulsion. Colored bands represent 95% confidence intervals

Seven bacterial strains belonging to different species typically found in cheese microbiomes were selected to evaluate the effect of invert emulsion on the growth of different bacteria. Namely, *Lactococcus lactis* subsp. *lactis* biovar diacetylactis, *Streptococcus thermophilus*, *Brevibacterium linens, Leuconostoc mesenteroides*, *Staphylococcus xylosus*, *Lactiplantibacillus plantarum* WHE92 and *Carnobacterium maltaromaticum* F2 (as a control) were cultivated separately in a classical setup or in invert emulsion on the course of 72 h incubation and enumerated each 24 h. Results show that strains exhibited different behaviors upon cultivation in invert emulsion (Fig. 6).

In the extremes, *C. maltaromaticum* and *S. xylosus* showed similar growth in invert emulsion compared to the classical setup, while *B. linens* did not grow. Interestingly, densities of *L. mesenteroides* and *L. plantarum* were similar after 72 h incubation in invert emulsion and in classical setup, but growth kinetics were slower and no decline phase was observed in invert emulsion. *L. lactis* and *S. thermophilus* were able to grow in invert emulsion, however the final densities in this system were between 1 and 2 log_10_ lower after cultivation with this method compared to the classical setup.

These results show that invert emulsion culture is not a neutral system for the growth of bacteria opening the possibility that this device can induce changes in structures of cultivated communities.

### Propagation of a bacterial consortium

**Fig. 7:**
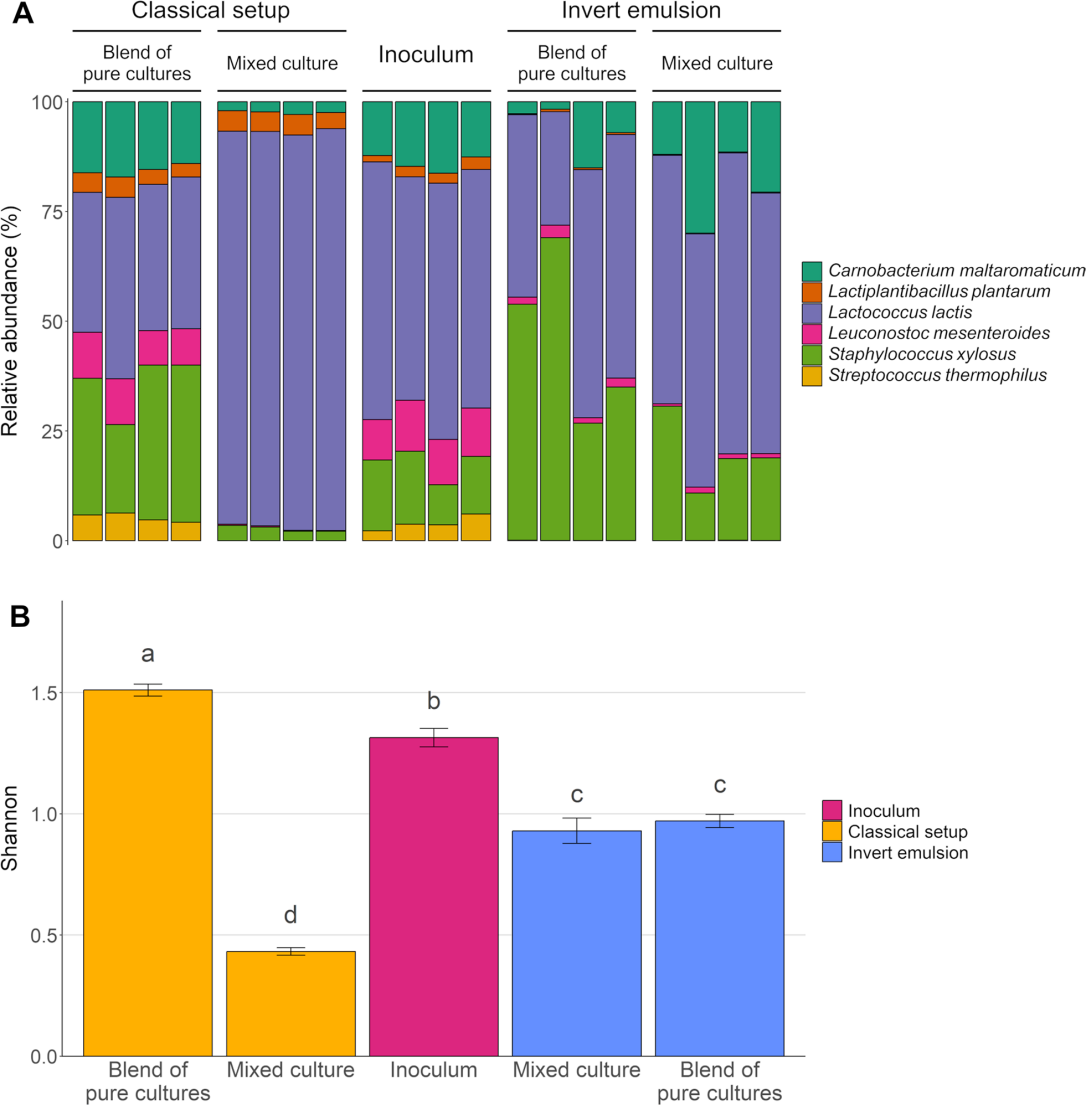
Communities propagated as mixed cultures or blends of separately grown bacteria in classical setup or invert emulsion. **A** Composition plots. Each individual bar corresponds to a replicate of the condition displayed in the stripe above. **B** Shannon diversity indices. Error bars represent the SEM and different superscript letters indicate statistically significant differences (*P* value < 0.05)

The six strains displaying growth in invert emulsion culture, i.e.,* Lactococcus lactis* subsp. *lactis* biovar diacetylactis, *Streptococcus thermophilus*, *Leuconostoc mesenteroides*, *Staphylococcus xylosus*, *Lactiplantibacillus plantarum* and *Carnobacterium maltaromaticum,* have been used to build a bacterial consortium in order to investigate the effect of the cultivation system on the community structure. The bacterial consortium was assembled by mixing equal volumes of overnight cultures of the strains at their optimal growth temperature (Table 1), and cultivated during 72 h at 30 °C as a mixed culture in a classical setup or in invert emulsion. In parallel, all strains were cultivated separately in each system for 72 h and were assembled by mixing equal volumes of cultures just before analysis. These blends of pure cultures were used to simulate the absence of interactions and to investigate the effect of each system on bacterial growth. The communities have been inoculated at approximately 6 log_10_ CFU mL^−1^ in order to avoid the entrapment of multiple bacteria in a single droplet of medium. Populations grew during incubation by a mean factor of 2.99 ± 0.09 log_10_ in classical setup and of 2.64 ± 0.04 log_10_ in invert emulsion. The structure of the communities was investigated by metabarcoding (Fig. 7A).

The Shannon alpha diversity index of the inoculum was 1.34 ± 0.04, and decreased to 0.46 ± 0.02, and 0.98 ± 0.03 after cultivation in a classical system and in invert emulsion, respectively (Fig. 7B). Thus, the diversity of the bacterial consortium significantly dropped during cultivation with both methods (*P* value < 0.001), but remained significantly higher after cultivation in invert emulsion (*P* value < 0.001). The low diversity observed in the community propagated in the classical setup can be attributable to *L. lactis* which dominated the cultivated community since 90.2% ± 0.5% of the sequencing reads were assigned to this species (Fig. 7A). As expected, no significant difference (*P* value ≥ 0.05) was found in invert emulsion between the Shannon indices of communities grown in mixed culture or in separate culture (blend). Thus, in invert emulsion culture, bacteria behaved in mixed culture as if they were cultivated separately. These results confirmed the absence of microbial interactions between the droplets of the emulsion, resulting in a higher diversity than in classical mixed culture.

**Fig. 8:**
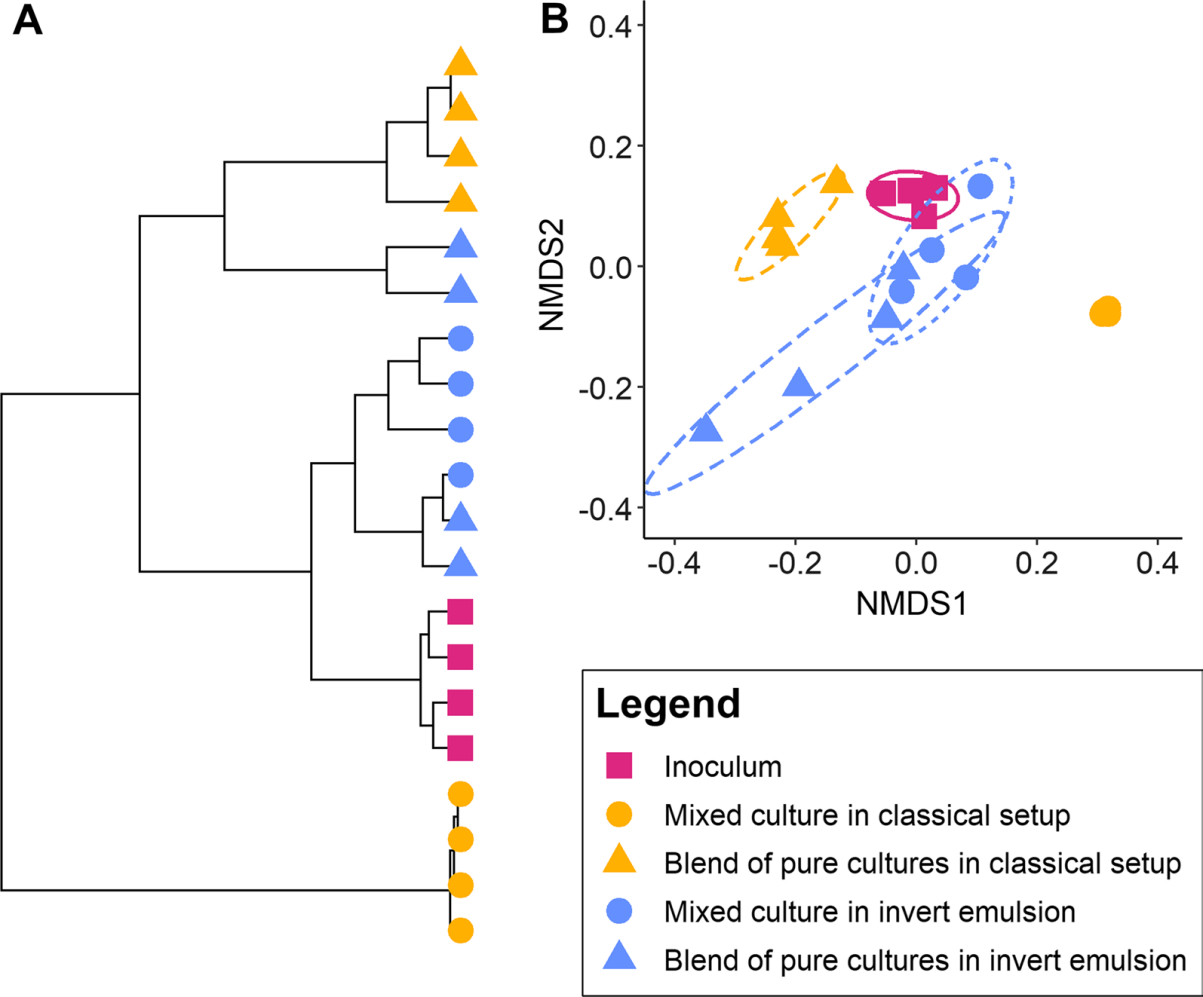
Clustering and ordination based on Bray–Curtis distances between samples. **A** Unsupervised clustering of samples using a centroid-based method (ward.D2). **B** Non-metric dimensional scaling (NMDS) of samples. Ellipses represent a 80% confidence level for a multivariate t-distribution. The legend is common to both panels

A clustering of the samples on the basis of Bray–Curtis distance matrix, and a centroid-based aggregation method, showed that mixed cultures performed in a classical setup were especially distinct from others, including the control blend of pure cultures in a classical setup (Fig. 8A).

This observation agrees with the ordination plot where these samples form a cluster apart from the others (Fig. 8B). As expected in the absence of microbial interactions, the clustering revealed that mixed and blends of emulsified cultures are intertwined (Fig. 8A), and in the ordination plot, both conditions are overlapping (Fig. 8B). Consistent with all preceding results, the structure of the communities cultivated in invert emulsion are closer to inocula and controls simulating the absence of interaction, than to the classical setup (Fig. 8). However, the samples from the mixed invert emulsion culture form a distinct cluster from the inoculum cluster (Fig. 8A). This result indicates that cultivation in invert emulsion result in a community characterized by a different structure. Indeed, the resulting communities have low relative abundance of *L. plantarum, L. mesenteroides,* and *S. thermophilus*, and reciprocally a high relative abundance of *S. xylosus* (Fig. 7A)*.*

Overall, these results show that invert emulsified culture medium can support growth of a bacterial consortium. Although microbial diversity decreases in all mixed culture conditions, the diversity remains higher in invert emulsion due to biotic confinement. In addition, the culture of communities in emulsified and non-emulsified medium leads to communities exhibiting distinct structures.

## Discussion

This study demonstrates that reproducible invert emulsions using culture medium could be easily obtained. In order to be suitable for bacterial cultivation, the invert emulsion must meet two requirements. Firstly, it had to be sufficiently stable during the growth of a given bacterium. Secondly, it had to contain droplets large enough to host and allow growth of bacteria, which size is generally comprised between 0.5 and 5 µm. In the system described here, most of the medium is dispersed into droplets of approximatively 40 µm diameter, which was deemed compatible with hosting and growth of bacteria. The introduction of bacteria has a negligible effect on the structure of the invert emulsion in small-scale systems. In addition, 24 h incubation does not significantly alter the structure of the emulsion. In bioreactor however, both introduction of bacteria and incubation time displace the distribution towards lower diameters, approximatively 25 µm. Nonetheless, these diameters are still compatible with hosting and growth of bacteria as shown by bioreactor pure and mixed culture experiments.

The invert emulsions used in this study consist of a culture medium dispersed in a continuous oil phase. CLSM observations showed that bacteria were located in aqueous droplets. The facultative anaerobic strain *C. maltaromaticum* F2 and the microaerophilic *L. monocytogenes* EGDe were introduced separately in the medium before emulsification. Bacterial enumerations showed that both strains can grow significantly (up to approximatively 3 log_10_) in invert emulsions up to 10^7^ CFU mL^−1^ inoculation levels, showing that this system can sustain bacterial growth. Lower final densities were observed in invert emulsion compared to a non-emulsified culture despite having reached the carrying capacity as shown by the analysis of kinetics. Regarding growth capacity during a 24 h incubation period, similar patterns have been observed for the six strains selected, with lower final densities in invert emulsion system. Cultivation upon a longer period (72 h) revealed that the cultivation device affected the growth of these bacteria, by diminishing the growth rate or the final density depending on the bacterium. The lower carrying capacity of the system might be inherent to the confinement of bacteria in droplets. Indeed, some droplets did not contain bacteria, therefore these droplets contained broth that was not available for bacterial growth. Another hypothesis is that this system could be highly sensitive to growth heterogeneity as it was observed for *L. lactis* [6]. This assumption is consistent with the fact that microdroplet technologies have been increasingly used for single-cell studies and clone selection [5, 34]. Other authors have shown that the confinement of entomopathogenic fungi and microalgae in droplets of invert emulsions dramatically increased their viability, therefore suggesting some sort of physiological changes in the encapsulated microorganisms [35]. Another hypothesis could be that growth into confined spaces could lead to inhibition due to the local density of bacteria within droplets, mediated by contact dependent or independent mechanisms [36]. Also, the absence of growth in *B. linens* in invert emulsion could be due to limitation in the oxygen supply. Indeed, all strains that were able to grow are facultative anaerobic bacteria while *B. linens* is strictly aerobic, thus suggesting that the invert emulsion cultivation technique is not suitable for the culture of strictly aerobic bacteria.

This work also demonstrated that bulk invert emulsions could be used as a culture system to improve the co-cultivation of antagonistic bacteria. This system relies on the spatial segregation of bacteria. The antagonism of the investigated bacterial model couple relies on the production of diffusible inhibitory compound, therefore focusing on interference competition. Yet, it is likely that the structure of the invert emulsion might also limit the effects of competition resulting from the exploitation of resources. Indeed, droplets of culture broth can be considered as microreactors enabling each bacterium to grow separately from each other, a structure that was supported by CLSM observations. It is reasonable to assume that the more bacteria are inoculated before emulsification, the more droplets are occupied and eventually co-occupied by different strains. This problem has long been recognized in the use of microfluidic devices and has been addressed by different authors on the basis of statistical modelling [37, 38] and experimentation [39]. While such models are especially useful for controlling cell encapsulation in invert emulsions produced by microfluidics, they remain challenging in the case of bulk invert emulsions due to their polydispersity.

The results presented here showed that there is a range of inoculation levels that are suitable to alleviate competition in this binary model of antagonism. In binary emulsified co-culture with a cell-to-cell ratio of 1:1, the optimal inoculation level should also not exceed 10^7^ CFU mL^−1^, otherwise the degree of co-occupation of droplets by antagonistic bacteria likely increases and competitive interactions can take place. These inoculum conditions were used to cultivate a bacterial consortium. There was no significant difference of alpha diversity between mixed emulsified cultures and blends of emulsified cultures. In addition, clustering analysis based on beta diversity revealed that both conditions were intertwined, and furthermore both conditions were not distinguishable by NMDS. Yet, the bacteria of the consortium are able to interact since the culture in the classical setup led to a community which derived strongly from the initial community. Therefore, bacteria in emulsified medium behave as if they were free of microbial interactions, which is in agreement with the data obtained with the two-strain antagonistic model which showed that emulsion could mitigate interactions such as interference competition. Although this study focused on competition, it could be assumed that invert emulsions could also alleviate other type of interactions such as mutualistic interactions. Indeed, a similarly structured system produced by microfluidics has been used in order to infer complex microbial interactions networks, involving mutualistic interactions [40]. Moreover, the present study focused on interspecific competition. Yet, it is reasonable to assume that it could also alleviate competition on an intraspecific level. Such results were described in experimental evolution experiments conducted by Bachmann et al. [6]. By lifting intraspecific competition, bulk emulsions could potentially allow the production of highly diversified communities of the same species, a sought-after property for starter cultures in the dairy industry [41].

Although the invert emulsion system allowed to alleviate microbial interactions, the community structure obtained after cultivation was different from the initial bacterial consortium. This is probably due to the effect of the system itself on the growth capacity of the microorganisms. Indeed, monitoring the growth of each microorganism in pure emulsified culture revealed its impact on the physiology of microorganisms. For instance, *B. linens* was not able to grow in this system, *L. plantarum* had a lower growth rate, *L. lactis* and *S. thermophilus* produced a lower biomass. Moreover, investigation of the effect of inoculation levels on the final bacterial densities in invert emulsion culture revealed that this system is sensitive to the initial bacterial densities. A low initial density leads to a low final density as access to the substrate for bacterial growth is likely to be limited by the compartmentation of the medium in invert emulsion. These different growth behaviors likely led to these divergent communities and opens the possibility of using invert emulsion not only to propagate communities but also as a tool to reshape them. This suggests that the present co-culture system could fit in the microbial community engineering toolbox [3, 42].

## Conclusions

This work demonstrates that invert emulsions containing bacteria can be obtained in a single step using regular emulsification methods. The invert emulsion system provides a substantial number of droplets with a mean diameter of approximately 40 µm that can harbor single bacteria and allow their growth. More importantly, bacteria are spatially segregated within this cultivation device, a property that leads to the reduction of bacterial competition in a binary bacterial model demonstrating strong antagonism in a classical culture device. In order to benefit from the alleviation of competition, this system should be inoculated with less than 10^7^ CFU mL^−1^. More specifically, the authors recommend that the bacterial mixture is inoculated at approximately 10^6^ CFU mL^−1^ so as to provide an optimal compromise between growth dynamics and alleviation of competition. Indeed, higher inoculation levels lead to lower growth dynamics and the loss of the latter property, likely resulting from the co-localization of antagonistic bacteria within the same droplets. In the propagation of a bacterial consortium, invert emulsions produced more diversified communities than non-emulsified medium, while being more conservative than a classical setup regarding structure. Bulk emulsions can also be prepared within standard bioreactors, fostering their use for industrial purposes. In a broader perspective, invert emulsion culture media represent an important potential for research on microbiota and for their valorization. Their use to limit exploitation and interference competition between microorganisms could be an important new tool for the production of innovations in the field of microbial community engineering.

## Supplementary Information

**Additional file 1: Figure S1.** Normalized excitation and emission spectra of E. coli DH5-α pGLO and Nile Red determined by scanning. Solid lines: excitation spectra; dotted lines: emission spectra; green: *E. coli* DH5-α pGLO; red: Nile Red. **Figure S2.** Double layer agar assay. One colony of *C. maltaromaticum* F2 was deposited in the center of the agar. After a first incubation, a second layer of agar containing *L. monocytogenes* EGDe was poured onto the first layer and the plate was incubated once again. **Figure S3.** Particle size distribution obtained by granulomorphometric analysis of emulsions in bioreactor. Logarithmic density volumetric distribution q3lg without bacteria (left) and with bacteria (right), respectively. Solid line, filled triangle: after emulsification; dashed line, empty circle: after 24 h incubation. Error bars represent the SEM. **Table S1.** Effect of inoculum levels on the growth of *C. maltaromaticum* F2 and *L. monocytogenes* EGDe in pure cultures using a classical setup or the invert emulsion system. Results are expressed as means ± SEM, fold change refers to the ratio of the final population to the initial population. **Table S2.** Effect of inoculum levels on the competition between *C. maltaromaticum* F2 and *L. monocytogenes* EGDe in mixed cultures using a classical setup or the invert emulsion system. Results are expressed as means ± SEM, fold change refers to the ratio of the final population to the initial population. **Figure S4.** Halo inhibition assay. The samples were deposited into wells previously dug in an agar layer inoculated with *L. monocytogenes* EGDe. **(**A) supernatant of a non-emulsified culture of *C. maltaromaticum* F2; (B) aqueous phase of an emulsified culture of *C. maltaromaticum* F2; (C) unaltered emulsified culture of *C. maltaromaticum* F2.

## Data Availability

The datasets presented in this study can be found in the data repository DOREL (Données de Recherche Lorraines) at https://doi.org/10.12763/7QIYMF
