# Supplementary material for: Invert emulsions alleviate biotic interactions in bacterial mixed culture
Source: Microb Cell Fact. 2023 Jan 20;22:16. doi: 10.1186/s12934-022-02014-w (PMC9854087; doi:10.1186/s12934-022-02014-w)
Supplement: Supplementary file 1 — Additional file 1: Figure S1. Normalized excitation and emission spectra of E. coli DH5-α pGLO and Nile Red determined by scanning. Solid lines: excitation spectra; dotted lines: emission spectra; green: E. coli DH5-α pGLO; red: Nile Red. Figure S2. Double layer agar assay. One colony of C. maltaromaticum F2 was deposited in the center of the agar. After a first incubation, a second layer of agar containing L. monocytogenes EGDe was poured onto the first layer and the plate was incubated once again. Figure S3. Particle size distribution obtained by granulomorphometric analysis of emulsions in bioreactor. Logarithmic density volumetric distribution q3lg without bacteria (left) and with bacteria (right), respectively. Solid line, filled triangle: after emulsification; dashed line, empty circle: after 24 h incubation. Error bars represent the SEM. Table S1. Effect of inoculum levels on the growth of C. maltaromaticum F2 and L. monocytogenes EGDe in pure cultures using a classical setup or the invert emulsion system. Results are expressed as means ± SEM, fold change refers to the ratio of the final population to the initial population. Table S2. Effect of inoculum levels on the competition between C. maltaromaticum F2 and L. monocytogenes EGDe in mixed cultures using a classical setup or the invert emulsion system. Results are expressed as means ± SEM, fold change refers to the ratio of the final population to the initial population. Figure S4. Halo inhibition assay. The samples were deposited into wells previously dug in an agar layer inoculated with L. monocytogenes EGDe. (A) supernatant of a non-emulsified culture of C. maltaromaticum F2; (B) aqueous phase of an emulsified culture of C. maltaromaticum F2; (C) unaltered emulsified culture of C. maltaromaticum F2. [file 12934_2022_2014_MOESM1_ESM.docx]

Additional file 1

# Excitation and emission spectra of *E. coli* DH5-α pGLO and Nile Red

**Figure S1. Normalized excitation and emission spectra of E. coli DH5-α pGLO and Nile Red determined by scanning.** Solid lines: excitation spectra; dotted lines: emission spectra; green: *E. coli* DH5-α pGLO; red: Nile Red.

Excitation (ex) and emission (em) spectra of the GFP-producing bacterium and lipophilic stain Nile Red were determined separately by scanning a broad range of wavelengths (λ) using CLSM. Results show that the maximum λ_ex_(GFP) was 481 nm, λ_em_(GFP) was in the range of 499 nm to 517 nm. The maximum λ_ex_(Nile Red) was 539 nm and λ_em_(Nile Red) was comprised between 559 nm and 613 nm (Figure S1).

# Double layer agar assay


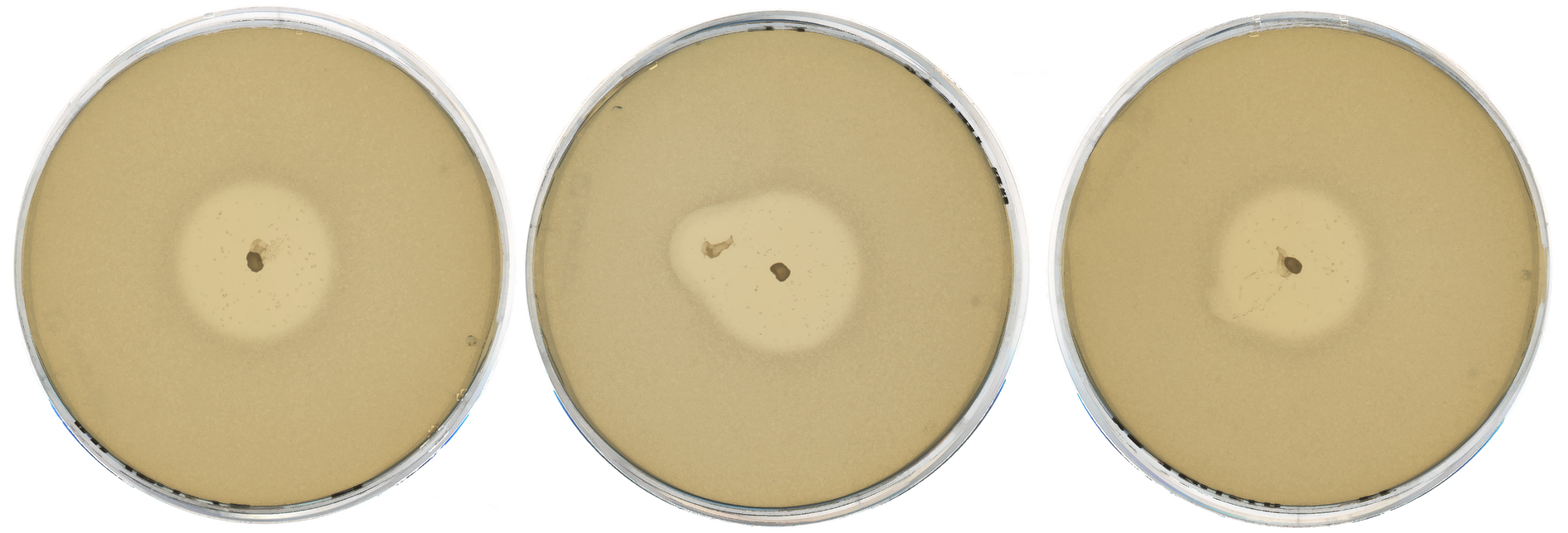


**Figure S2. Double layer agar assay.** One colony of *C. maltaromaticum* F2 was deposited in the center of the agar. After a first incubation, a second layer of agar containing *L. monocytogenes* EGDe was poured onto the first layer and the plate was incubated once again.

After a second incubation, the agar became turbid except in an area surrounding the colony, indicating inhibition (Figure S2).

# Bioreactor-scale invert emulsions


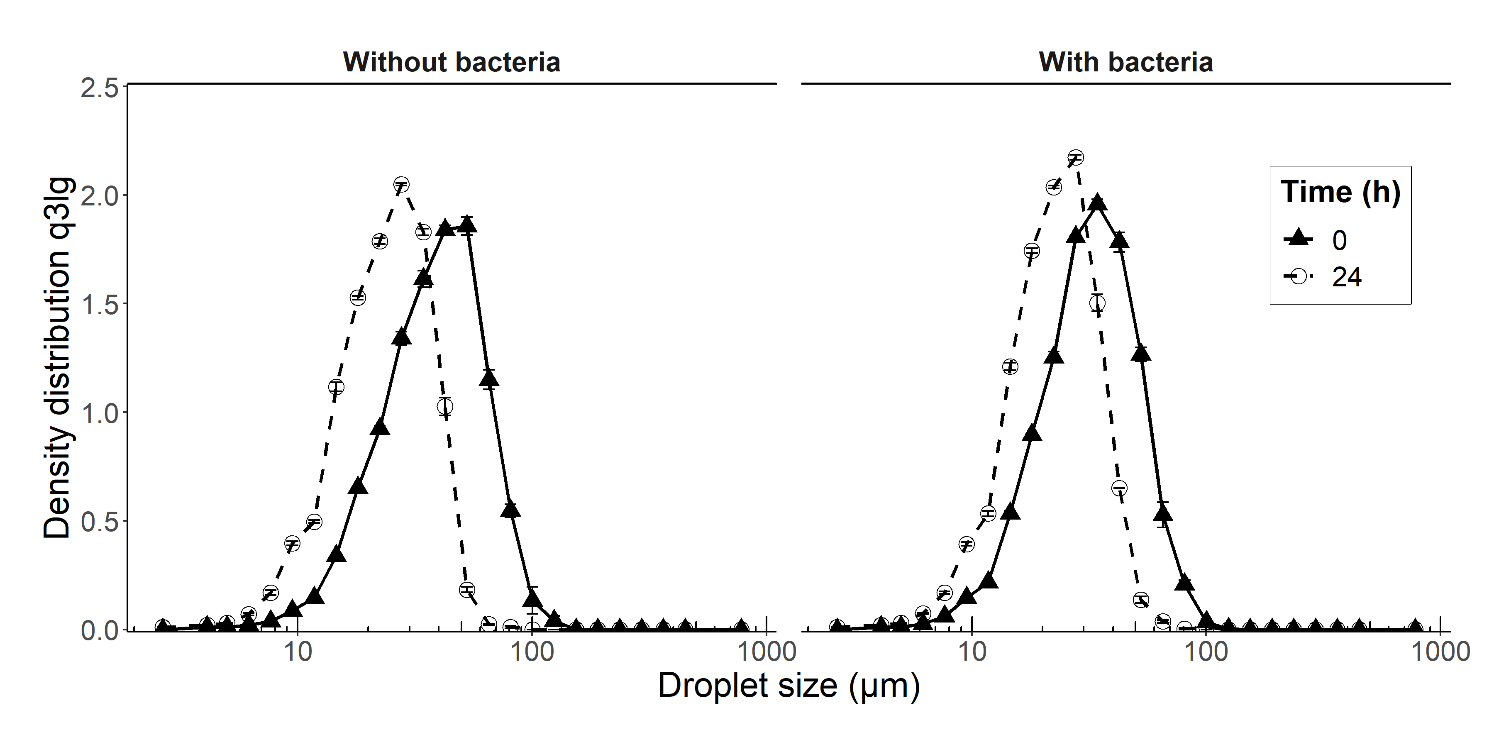


**Figure S3. Particle size distribution obtained by granulomorphometric analysis of emulsions in bioreactor.** Logarithmic density volumetric distribution q3lg without bacteria (left) and with bacteria (right), respectively. Solid line, filled triangle: after emulsification; dashed line, empty circle: after 24 h incubation. Error bars represent the SEM.

The invert emulsion culture system was scaled-up to 600 mL for demonstration. Particle size distributions were obtained by granulomorphometric analysis using the QICPIC device (Figure S3). Results are described in section 3.4 of manuscript.

# Effect of inoculum levels on bacterial growth

**Table S1. Effect of inoculum levels on the growth of *C. maltaromaticum* F2 and *L. monocytogenes* EGDe in pure cultures using a classical setup or the invert emulsion system.** Results are expressed as means ± SEM, fold change refers to the ratio of the final population to the initial population.

| **Scale** | **Strain** | **System** | **Initial population (log_10_ CFU.mL^-1^)** | **Final population (log_10_ CFU.mL^-1^)** | **Fold change (log_10_)** |
| --- | --- | --- | --- | --- | --- |
| Small-scale | *C. maltaromaticum* F2 | Classical setup | 2.84 ± 0.08 | 9.09 ± 0.03 | 6.25 ± 0.06 |
|  |  |  | 3.93 ± 0.05 | 9.10 ± 0.03 | 5.17 ± 0.07 |
|  |  |  | 4.97 ± 0.06 | 9.09 ± 0.04 | 4.12 ± 0.07 |
|  |  |  | 6.01 ± 0.02 | 9.26 ± 0.06 | 3.25 ± 0.07 |
|  |  |  | 7.01 ± 0.06 | 9.09 ± 0.02 | 2.08 ± 0.05 |
|  |  |  | 7.97 ± 0.06 | 9.14 ± 0.14 | 1.16 ± 0.08 |
|  |  | Invert emulsion | 3.15 ± 0.24 | 5.42 ± 0.29 | 2.28 ± 0.48 |
|  |  |  | 4.12 ± 0.01 | 6.34 ± 0.47 | 2.22 ± 0.48 |
|  |  |  | 5.02 ± 0.07 | 7.64 ± 0.27 | 2.62 ± 0.21 |
|  |  |  | 5.86 ± 0.02 | 8.50 ± 0.11 | 2.64 ± 0.12 |
|  |  |  | 7.07 ± 0.07 | 9.01 ± 0.06 | 1.94 ± 0.11 |
|  |  |  | 7.85 ± 0.12 | 8.47 ± 0.22 | 0.62 ± 0.11 |
|  | *L. monocytogenes* EGDe | Classical setup | 3.13 ± 0.12 | 9.02 ± 0.25 | 5.89 ± 0.37 |
|  |  |  | 3.96 ± 0.06 | 8.97 ± 0.16 | 5.02 ± 0.11 |
|  |  |  | 5.21 ± 0.09 | 8.77 ± 0.16 | 3.57 ± 0.24 |
|  |  |  | 6.24 ± 0.08 | 9.06 ± 0.12 | 2.82 ± 0.19 |
|  |  |  | 7.17 ± 0.09 | 9.07 ± 0.23 | 1.91 ± 0.25 |
|  |  |  | 8.09 ± 0.05 | 8.85 ± 0.13 | 0.76 ± 0.18 |
|  |  | Invert emulsion | 3.24 ± 0.30 | 5.76 ± 0.12 | 2.52 ± 0.18 |
|  |  |  | 4.10 ± 0.11 | 6.87 ± 0.07 | 2.77 ± 0.08 |
|  |  |  | 5.19 ± 0.13 | 8.16 ± 0.38 | 2.97 ± 0.29 |
|  |  |  | 6.06 ± 0.07 | 8.82 ± 0.19 | 2.76 ± 0.18 |
|  |  |  | 7.24 ± 0.04 | 8.88 ± 0.14 | 1.64 ± 0.10 |
|  |  |  | 7.79 ± 0.23 | 8.86 ± 0.09 | 1.07 ± 0.31 |
| Bioreactor | *C. maltaromaticum* F2 | Classical setup | 5.75 | 8.54 | 2.78 |
|  |  | Invert emulsion | 5.90 | 8.63 | 2.73 |
|  | *L. monocytogenes* EGDe | Classical setup | 6.22 | 8.24 | 2.02 |
|  |  | Invert emulsion | 6.17 | 8.41 | 2.24 |

# Effect of inoculum levels on bacterial competition

**Table S2. Effect of inoculum levels on the competition between *C. maltaromaticum* F2 and *L. monocytogenes* EGDe in mixed cultures using a classical setup or the invert emulsion system.** Results are expressed as means ± SEM, fold change refers to the ratio of the final population to the initial population.

| **Scale** | **System** | **Strain** | **Initial population (log_10_ CFU.mL^-1^)** | **Final population**  **(log_10_ CFU.mL^-1^)** | **Fold change (log_10_)** |
| --- | --- | --- | --- | --- | --- |
| Small-scale | Classical setup | *C. maltaromaticum* F2 | 2.70 ± 0.20 | 9.19 ± 0.09 | 6.49 ± 0.13 |
|  |  | *L. monocytogenes* EGDe | 3.01 ± 0.11 | 6.10 ± 0.05 | 3.08 ± 0.16 |
|  |  | *C. maltaromaticum* F2 | 3.92 ± 0.11 | 9.08 ± 0.03 | 5.16 ± 0.15 |
|  |  | *L. monocytogenes* EGDe | 3.99 ± 0.03 | 6.29 ± 0.13 | 2.30 ± 0.14 |
|  |  | *C. maltaromaticum* F2 | 4.93 ± 0.12 | 9.09 ± 0.08 | 4.17 ± 0.13 |
|  |  | *L. monocytogenes* EGDe | 5.14 ± 0.05 | 6.62 ± 0.06 | 1.48 ± 0.11 |
|  |  | *C. maltaromaticum* F2 | 5.92 ± 0.14 | 9.35 ± 0.07 | 3.42 ± 0.19 |
|  |  | *L. monocytogenes* EGDe | 6.00 ± 0.11 | 5.52 ± 0.20 | -0.48 ± 0.11 |
|  |  | *C. maltaromaticum* F2 | 6.87 ± 0.09 | 9.12 ± 0.09 | 2.25 ± 0.04 |
|  |  | *L. monocytogenes* EGDe | 6.98 ± 0.21 | 5.48 ± 0.09 | -1.50 ± 0.21 |
|  |  | *C. maltaromaticum* F2 | 7.66 ± 0.11 | 8.98 ± 0.13 | 1.31 ± 0.13 |
|  |  | *L. monocytogenes* EGDe | 8.01 ± 0.04 | 5.00 ± 0.15 | -3.00 ± 0.13 |
|  | Invert emulsion | *C. maltaromaticum* F2 | 2.76 ± 0.03 | 5.18 ± 0.12 | 2.41 ± 0.10 |
|  |  | *L. monocytogenes* EGDe | 2.97 ± 0.16 | 5.12 ± 0.06 | 2.15 ± 0.10 |
|  |  | *C. maltaromaticum* F2 | 4.03 ± 0.08 | 5.94 ± 0.27 | 1.91 ± 0.27 |
|  |  | *L. monocytogenes* EGDe | 4.06 ± 0.09 | 6.24 ± 0.18 | 2.18 ± 0.09 |
|  |  | *C. maltaromaticum* F2 | 4.77 ± 0.13 | 7.04 ± 0.06 | 2.27 ± 0.18 |
|  |  | *L. monocytogenes* EGDe | 5.13 ± 0.15 | 7.68 ± 0.07 | 2.55 ± 0.22 |
|  |  | *C. maltaromaticum* F2 | 5.85 ± 0.09 | 8.30 ± 0.13 | 2.45 ± 0.17 |
|  |  | *L. monocytogenes* EGDe | 5.96 ± 0.07 | 8.36 ± 0.12 | 2.40 ± 0.08 |
|  |  | *C. maltaromaticum* F2 | 6.99 ± 0.19 | 8.42 ± 0.28 | 1.43 ± 0.42 |
|  |  | *L. monocytogenes* EGDe | 7.06 ± 0.07 | 8.43 ± 0.17 | 1.37 ± 0.21 |
|  |  | *C. maltaromaticum* F2 | 7.95 ± 0.05 | 8.62 ± 0.27 | 0.67 ± 0.27 |
|  |  | *L. monocytogenes* EGDe | 7.83 ± 0.12 | 5.23 ± 0.11 | -2.60 ± 0.23 |
| Bioreactor | Classical setup | *C. maltaromaticum* F2 | 5.77 ± 0.18 | 8.47 ± 0.08 | 2.70 ± 0.25 |
|  |  | *L. monocytogenes* EGDe | 6.31 ± 0.05 | 6.06 ± 0.06 | -0.25 ± 0.01 |
|  | Invert emulsion | *C. maltaromaticum* F2 | 5.97 ± 0.09 | 8.22 ± 0.09 | 2.25 ± 0.17 |
|  |  | *L. monocytogenes* EGDe | 5.83 ± 0.05 | 8.11 ± 0.29 | 2.28 ± 0.24 |

# Halo inhibition assay

**
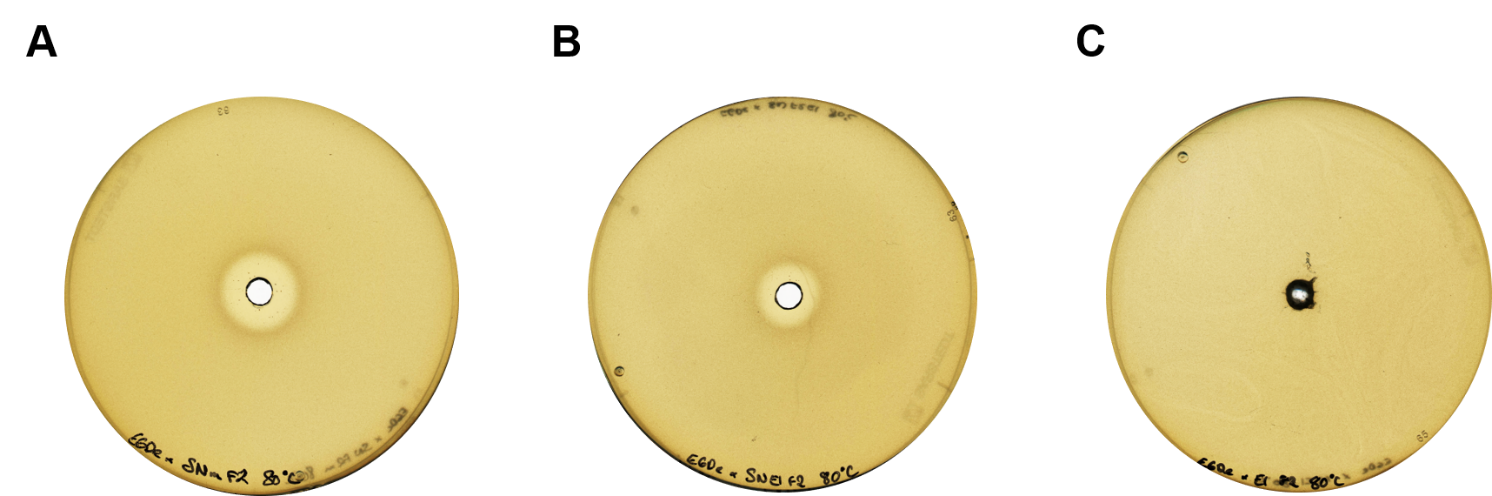
**

**Figure S4. Halo inhibition assay.** The samples were deposited into wells previously dug in an agar layer inoculated with *L. monocytogenes* EGDe. **(**A) supernatant of a non-emulsified culture of *C. maltaromaticum* F2; (B) aqueous phase of an emulsified culture of *C. maltaromaticum* F2; (C) unaltered emulsified culture of *C. maltaromaticum* F2.

# Data availability

The datasets presented in this study can be found in the data repository DOREL (Données de la Recherche Lorraines) at <https://doi.org/10.12763/7QIYMF>
